# Pathological classification of human iPSC-derived neural stem/progenitor cells towards safety assessment of transplantation therapy for CNS diseases

**DOI:** 10.1186/s13041-016-0265-8

**Published:** 2016-09-19

**Authors:** Keiko Sugai, Ryuji Fukuzawa, Tomoko Shofuda, Hayato Fukusumi, Soya Kawabata, Yuichiro Nishiyama, Yuichiro Higuchi, Kenji Kawai, Miho Isoda, Daisuke Kanematsu, Tomoko Hashimoto-Tamaoki, Jun Kohyama, Akio Iwanami, Hiroshi Suemizu, Eiji Ikeda, Morio Matsumoto, Yonehiro Kanemura, Masaya Nakamura, Hideyuki Okano

**Affiliations:** 1 Department of Orthopaedic Surgery, Keio University School of Medicine, Shinjuku, Tokyo, 160-8582 Japan; 2 Department of Physiology, Keio University School of Medicine, 35 Shinanomachi, Shinjuku, Tokyo, 160-8582 Japan; 3 Department of Pathology, Tokyo Metropolitan Children’s Medical Center, Fuchu, Tokyo, 183-8561 Japan; 4 Division of Stem Cell Research, Institute for Clinical Research, Osaka National Hospital, National Hospital Organization, Chuo-ku, Osaka, 540-0006 Japan; 5 Division of Regenerative Medicine, Institute for Clinical Research, Osaka National Hospital, National Hospital Organization, Chuo-ku, Osaka, 540-0006 Japan; 6 Central Institute for Experimental Animals, Kawasaki, Kanagawa 210-0821 Japan; 7 Regenerative & Cellular Medicine Office, Sumitomo Dainippon Pharma Co., Ltd., Kobe, Hyogo 650-0047 Japan; 8 Department of Genetics, Hyogo College of Medicine, Nishinomiya, Hyogo 663-8501 Japan; 9 Department of Pathology, Yamaguchi University Graduate School of Medicine, Ube, Yamaguchi 755-8505 Japan; 10 Department of Neurosurgery, Osaka National Hospital, National Hospital Organization, Chuo-ku, Osaka 540-0006 Japan

**Keywords:** Human induced pluripotent stem cells, Regenerative medicine, Cell transplantation, Carcinogenesis, Pathology

## Abstract

The risk of tumorigenicity is a hurdle for regenerative medicine using induced pluripotent stem cells (iPSCs). Although teratoma formation is readily distinguishable, the malignant transformation of iPSC derivatives has not been clearly defined due to insufficient analysis of histology and phenotype. In the present study, we evaluated the histology of neural stem/progenitor cells (NSPCs) generated from integration-free human peripheral blood mononuclear cell (PBMC)-derived iPSCs (iPSC-NSPCs) following transplantation into central nervous system (CNS) of immunodeficient mice. We found that transplanted iPSC-NSPCs produced differentiation patterns resembling those in embryonic CNS development, and that the microenvironment of the final site of migration affected their maturational stage. Genomic instability of iPSCs correlated with increased proliferation of transplants, although no carcinogenesis was evident. The histological classifications presented here may provide cues for addressing potential safety issues confronting regenerative medicine involving iPSCs.

**Electronic supplementary material:**

The online version of this article (doi:10.1186/s13041-016-0265-8) contains supplementary material, which is available to authorized users.

## Introduction

The transplantation of neural stem progenitor cells (NSPCs) is considered a promising approach to the treatment of a range of central nervous system (CNS) disorders, including spinal cord injury [1, 2], brain infarction [3–5], amyotrophic lateral sclerosis (ALS) [6, 7], and Parkinson’s disease [8]. In countries in which the clinical use of fetal NSPCs is not permitted due to ethical limitations, induced pluripotent stem cells (iPSCs) represent a potential alternative source of NSPCs for use in cell therapy research and development. In Japan, iPSC stocks [9, 10] are being established from peripheral blood mononuclear cells (PBMCs) of donors with a range of immunologically preferable genotypes. However, before such cells can be used in clinical applications, the safety of transplanted cells must be determined.

Transplant safety issues include infection, immunological problems such as rejection, complications resulting from drugs such as immunosuppressants, and complications resulting from unexpected migration or transplant behavior [11, 12]. As for stem cells, which have the potential to develop into a variety of mature tissue types, efforts must be made to manage the risk of contamination by undifferentiated pluripotent cells, or the transformation of graft-derived intermediate progenitors into malignant tumor cells [13–16].

Malignant tumors occasionally exhibit immature and embryonic-like structures, and normal embryonic cells share some characteristics with malignant tumor cells; i.e., normal developing tissues in which stem cell multiplication occurs exhibit significant mitotic activity, similar to that in malignant tumors. Under such conditions, the cellular mitotic index is not helpful in distinguishing stem cells from malignant tumors. NSPCs for transplantation therapies also exhibit characteristics of less developmentally mature cells, and thus it is necessary to classify the histology of transplanted cells by their developmental characteristics in order to distinguish them from the malignant transformation of transplants.

In the present study, we induced NSPCs from integration-free human peripheral blood mononuclear cell (PBMC)-derived iPSCs (iPSC-NSPCs) using two different protocols, compared their in vitro properties, and also compared their in vivo histology by transplanting them into intact striata or injured spinal cords of immunodeficient (NOD/Shi-*scid*, IL-2R γ null (NOG) [17] or NOD/*scid* [18]) mice. Our histological categorization may serve as a useful tool for predicting and describing the performance of NSPCs for future quality evaluations of cell products for future transplantation therapy.

## Results

### Induction of NSPCs from three human PBMC-derived iPSC lines

**Fig. 1:**
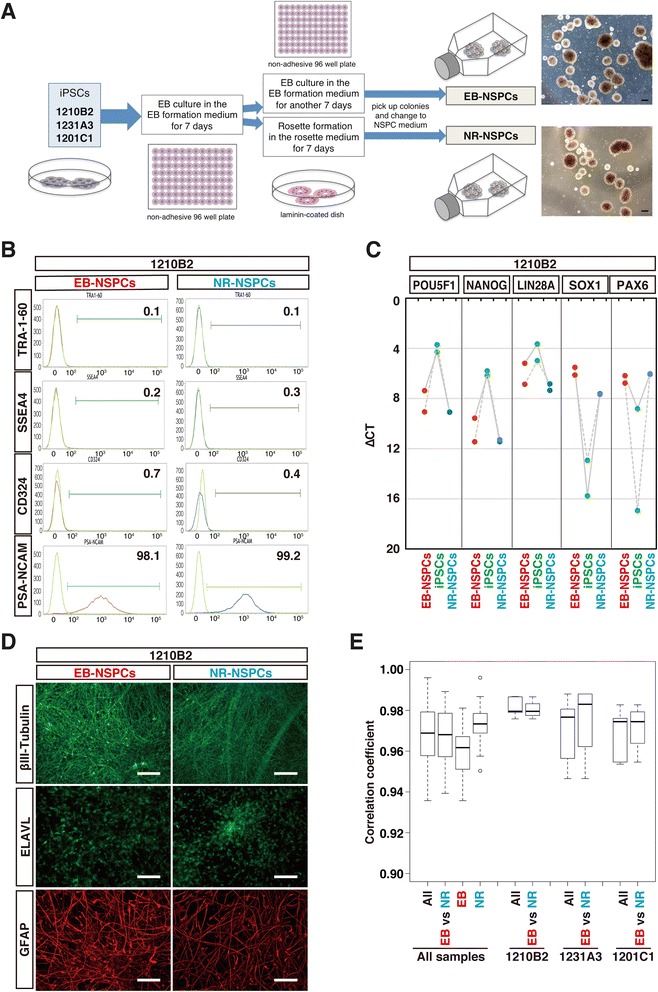
Schematic neural induction diagrams and characterization of the NSPCs generated from human PBMC-derived iPSCs. **a** Schematics of the NSPC induction protocols used in this study. (Scale = 200 μm for the images of neurospheres.) (**b**, **c**, **d**) Representative data taken by 1210B2-NSPCs for characterization analysis of the NSPCs. **b** Cell surface markers of the induced NSPCs. **c** The quantitative RT-PCR analysis results are depicted by ΔCt values. Quantitative RT − PCR analysis confirmed the decrease in the iPSC markers, and an increase in NSPC markers following the differentiation of iPSCs into NSPCs. (*n* = 2 for each iPSC-NSPC lines) **d** Representative immunocytochemistry data collected to confirm the differentiation potential of NSPCs into neuronal and glial lineages. (Scale = 100 μm.) **e** The correlation coefficients of the expression profiles among the NSPCs. The microarray profiles were similar between all the NSPCs regardless of the induction protocols or iPSCs. (*n* = 2 for each iPSC-NSPC lines) See also Additional file 1: Figure S1 and Additional file 2: Table S1

Three human integration-free iPSC lines made with episomal vectors (1210B2, 1231A3, and 1201C1) from the PBMC of single donor were differentiated into NSPCs by two protocols, which are easily modifiable into xeno-free protocols for clinical use (Fig. 1a). We refer to NSPCs induced directly from embryoid bodies (EBs) as EB-NSPCs, and those induced from the neural rosette (NR) phase as NR-NSPCs. Both EB-NSPCs and NR-NSPCs were expanded as free-floating neurospheres (Fig. 1a).

The differentiation of three iPSC clones into NSPCs was confirmed with flow cytometric analysis (Fig. 1b and Additional file 1: Figure S1A) in which low pluripotency marker expression (TRA-1-60, SSEA4, and CD324 (E-Cadherin) [19, 20]) and high neural marker expression (PSA-NCAM) [21] were observed. We also confirmed by RT-PCR that pluripotency marker expression (POUF5F1 (also called OCT4), NANOG, and LIN28A) decreased, and neural marker expression (SOX1 and PAX6) increased during their differentiation into NSPCs (Fig. 1c and Additional file 1: Figure S1B). Statistical analysis did not reveal any significant differences between EB- and NR-NSPCs. The differentiation potential of the three iPSC-derived NSPC clones as classical neural progenitors was confirmed by induction into neuronal (βIII-tubulin, ELAVL) and glial (GFAP) lineages by immunocytochemistry (Fig. 1d and Additional file 1: Figure S1C). All of the above assays were performed, and successful neural differentiation was confirmed at passage 6 (EBs) or 7 (NRs) of the NSPCs, suggesting that all three integration-free human PBMC-derived iPSCs examined had been induced to differentiate into NSPCs under both protocols via passage 7. We further characterized and compared the properties of each induced NSPCs by microarray expression analysis and found that all EB- and NR-NSPCs from the same iPSCs had profiles that closely resembled each other, with correlations of > 97.3 % (Fig. 1e and Additional file 2: Table S1). These results suggest that the NSPCs we established using two independent protocols had similar transcriptome properties. Correlation analyses among the NSPCs also indicated that 1210B2 iPSCs could be stably induced into NSPCs with the highest homogeneity; however, the results of our statistical analysis did not exceed the significance threshold (Fig. 1e).

### Abnormal karyotype and genomic instability in iPSCs result in altered NSPC proliferative capacities in vitro

**Fig. 2:**
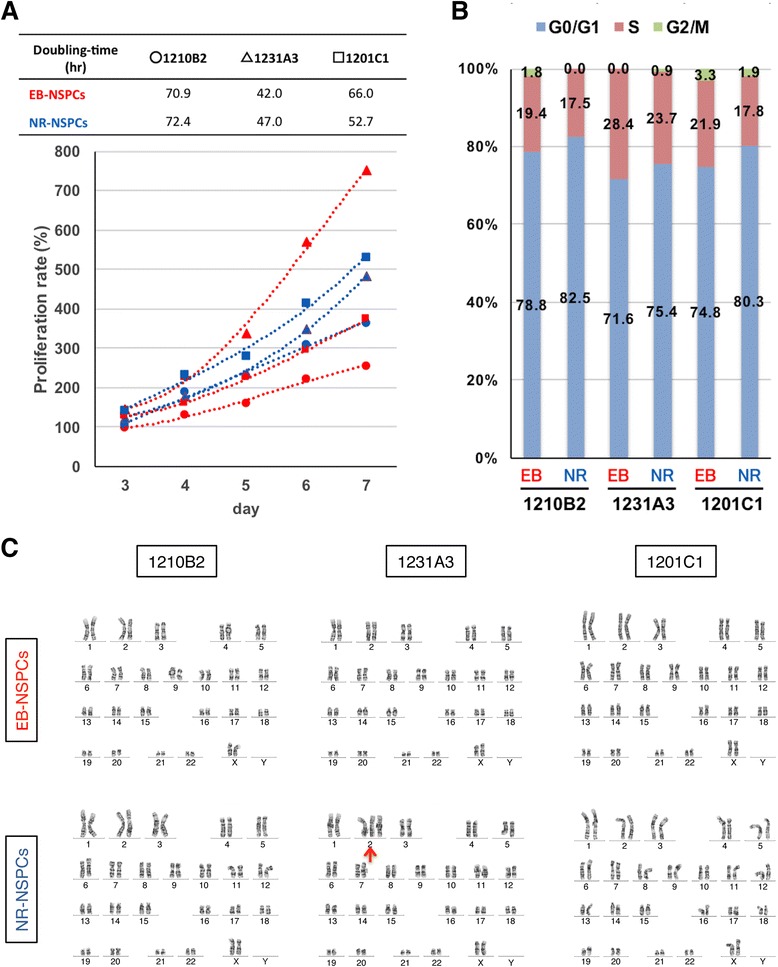
In vitro abnormality analysis of the induced NSPCs. Karyotype abnormalities were seen in the 1231A3 NSPCs and CNV abnormalities were seen in the 1231A3 and 1201C1 NSPCs. **a** Cellular doubling times were calculated by measuring ATP consumption for each of the NSPC lines. **b** The cell cycle for each NSPC cell line. Although a higher percentage of 1231A3-NSPCs were in S phase, there was no significant difference between the cell lines. **c** Results of karyotype analysis by G band-staining are shown. Only the 1231A3 NR-NSPCs had a karyotype abnormality. See also Additional file 4: Figure S2, and Additional file 5: Tables S3, Additional file 6: Table S4, Additional file 7: Table S5 and Additional file 8: Table S6

To examine the quality of the NSPCs, we evaluated their proliferation ratios (Fig. 2a). All cells analyzed showed consistent proliferative properties and could be maintained for more than 70 days. Of the cell lines compared, the NR-NSPCs had a proliferation ratio similar to that of the EB-NSPCs. Cellular doubling time was faster in the 1231A3 EB-NSPCs and slower in the 1210B2 EB-NSPCs and 1210B2 NR-NSPCs compared with the other NSPCs (Fig. 2a). In a cell cycle analysis, 1231A3 NSPCs showed a lower ratio of cells in the G1/G0 phase, indicating the presence of a higher population in their proliferative state (Fig. 2b).

To gain more detailed insights into the different proliferative kinetics among the NSPCs, we sought to identify any genetic abnormalities in these cells. In a karyotype analysis, the 1231A3 NR-NSPCs met the abnormality criterion, showing a high ratio of gain of chromosome 2 (Fig. 2c and Additional file 3: Table S2). Furthermore, copy number variations (CNVs) in NSPCs at passages six and seven were compared with those of the source iPSCs and NSPCs cultured for an additional five passages. We found that the largest number of *de novo* CNVs during differentiation and neurosphere culture occurred in the 1231A3 NR-NSPCs, and that CNV frequency increased over the course of additional culture of five passages. No (1210B2 EB-NSPCs) or single (1210B2 NR-NSPCs) *de novo* CNV was found in the 1210B2-iPSC-derived NSPCs. A few CNVs were found in the 1201C1-iPSCs during neural induction; however, the 1201C1-NSPCs were maintained with a stable genome over 10 passages (Additional file 4: Figure S2, Additional file 5: Table S3, Additional file 6: Table S4, Additional file 7: Table S5 and Additional file 8: Table S6).

These results suggest that most induced NSPCs can be safely generated on a large scale for future commercial use; however, as in the case of 1231A3 NR-NSPCs, NSPCs may exhibit abnormal karyotypes, resulting in an inhomogeneous, and possibly a highly proliferative state. Furthermore, many *de novo* CNVs were found in NSPCs at passage 6 or 7, and accumulated along with the culture length in the 1231A3 NSPCs, but very few *de novo* CNVs were found in the 1201C1 NSPCs and 1210B2 NSPCs, suggesting that the genomic stability of the original iPSCs may contribute to genomic instability of their derivative NSPCs.

### Cells with a higher proliferation ratio in vitro formed larger tissues when transplanted into immunodeficient mice

**Fig. 3:**
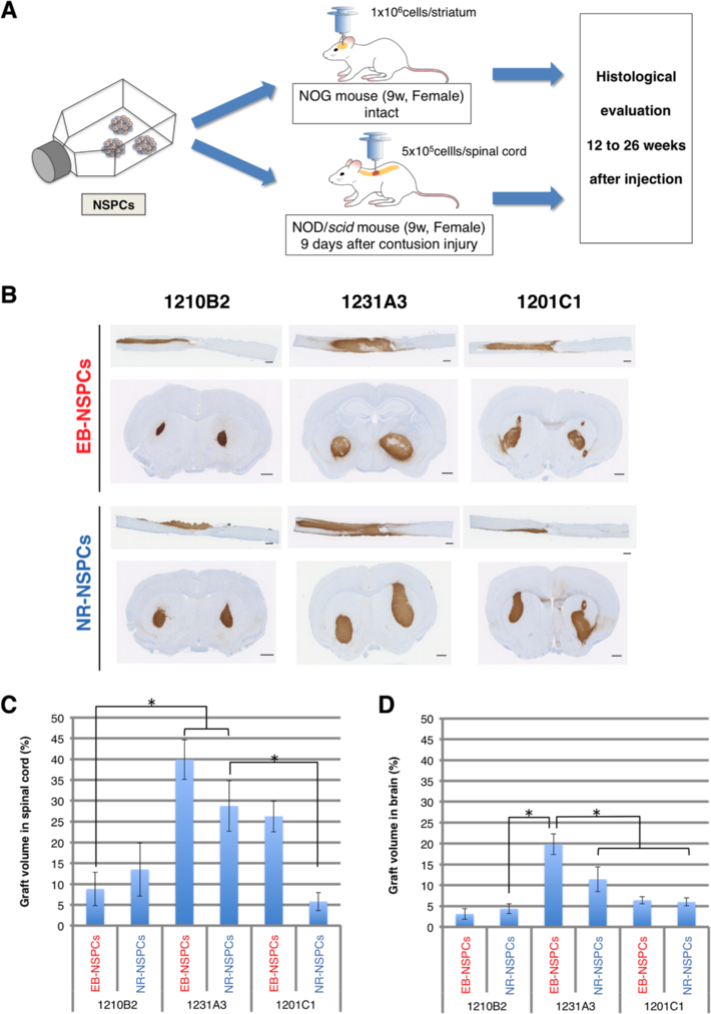
Histology revealed proliferative characteristics of the 1231A3-NSPCs both in intact brains and injured spinal cords. **a** Schematic of the in vivo transplantation protocol that was used. **b** Representative tissue sections of the spinal cord (upper row, 12 weeks after transplant) and brain (lower row, 26 weeks after transplant) after the transplantation of each cell line. Immunohistochemistry results for STEM121 and DAB, which were positive in the cytoplasm of the transplanted human cells. (Scale = 500 μm.) (**c**, **d**) The mean graft volume percentages in the injured spinal cord (**c**) and brain (**d**) sections are shown. Although the volumes were generally smaller in the brains compared with the injured spinal cords, the 1231A3 EB-NSPCs showed significantly greater proliferation levels in both the spinal cord and brain, and the 1231A3 NR-NSPCs also formed large tissues. Values are means ± SEM. *P < 0.05 (**c**: *n* = 4, 4, 4, 6, 3, 3. **d** *n* = 2, 3, 6, 6, 6, 6)

To further characterize NSPCs in vivo, we transplanted them into intact striata of NOG mice or into post-injured spinal cords of NOD/scid mice (Fig. 3a). Subsequent histological analyses were performed 12–26 weeks later by immunostaining with the human cytosol marker STEM121 [4, 22]. Cell engraftment patterns were similar to those of NSPCs derived from iPSCs generated from cells of different somatic origin (Additional file 9: Figure S3). The extent to which transplanted cells were distributed differed among the cell lines evaluated (Fig. 3bc and d). The 1231A3 NSPCs spread over larger areas, and the 1210B2 NSPCs spread over smaller areas, both in the injured spinal cord and in the brain (Fig. 3b, c and d). This trend was similar to the results of the in vitro proliferation analysis, suggesting that the cellular proliferation characteristics were maintained even after transplantation into mice.

### Terminal histology of transplanted NSPCs mimic a fetal neural differentiation pattern

**Fig. 4:**
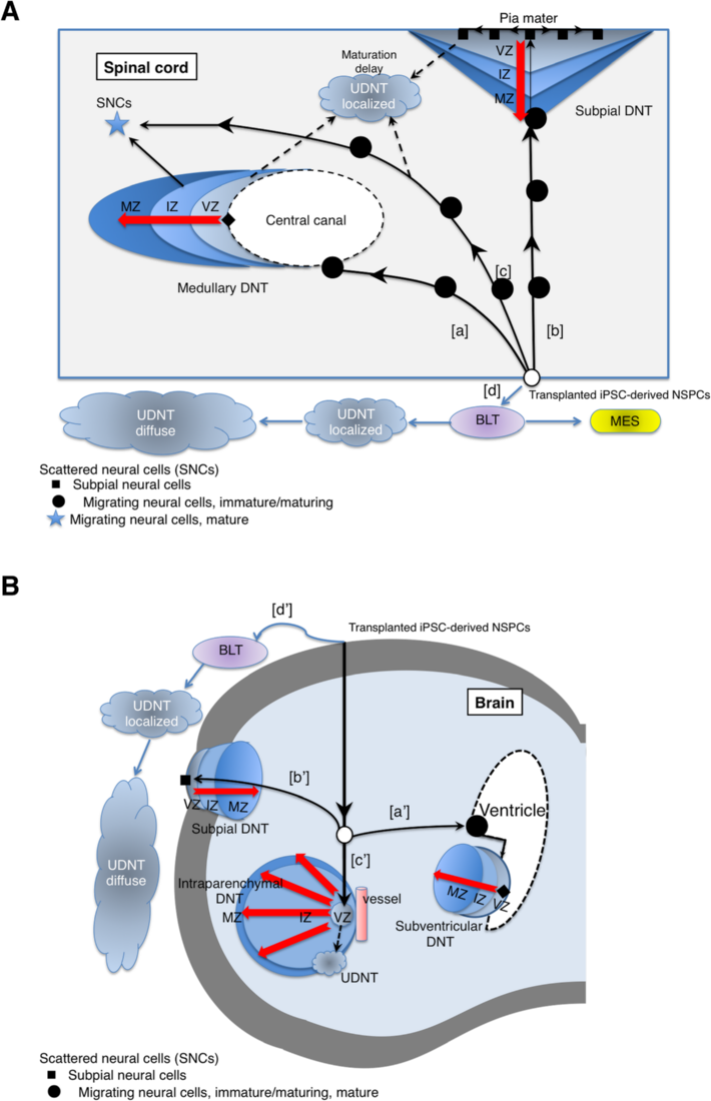
NSPC histology schematic in injured spinal cord and brain, according to the maturational pattern. **A**, **B** The transplant fate schematics in an injured spinal cord (**A**) and an intact brain (**B**). The transplanted cells reached the edge of a cyst that resembled the central canal in the injured spinal cords (**a**), the ventricle of brain (**a’**), the inner pia mater (**b**, **b’**), or vessels in the brain striatum (**c’**), which recapitulated fetal central nervous system development consisting of DNT. Medullary DNT arose from the edge of the central canal where transplanted cells transformed into neuroepithelium and neuroblasts (closed diamond), which gave rise to three cellular zones with different maturation patterns (ventricular zone (VZ)-, intermediate zone (IZ)-, and marginal zone (MZ)-like structures), as observed in fetal brain development. The migrating neural cells attached to the inner pia mater become flattened in shape (subpial neural cells, closed square), which had an equivalent immunophenotype to a VZ component of the DNT. UDNT are composed of cells that show a VZ equivalent immunophenotype, which are thought to be formed as the consequence of a maturational delay in the DNT formation process (broken arrows). **c** The migrating transplanted cells that did not reach the above neurogenesis initiation sites may differentiate into mature neural cells (scattered neural cells: SNC, indicated by a star) or fail to differentiate and may form a UDNT (broken arrow). SNCs might also be derived from DNT. **d**, **d’** The transplanted cells that failed to engraft into the parenchyma exhibit arrested or incomplete neural differentiation (BLT, UDNT) or mesenchymal differentiation (MES)

**Fig. 5:**
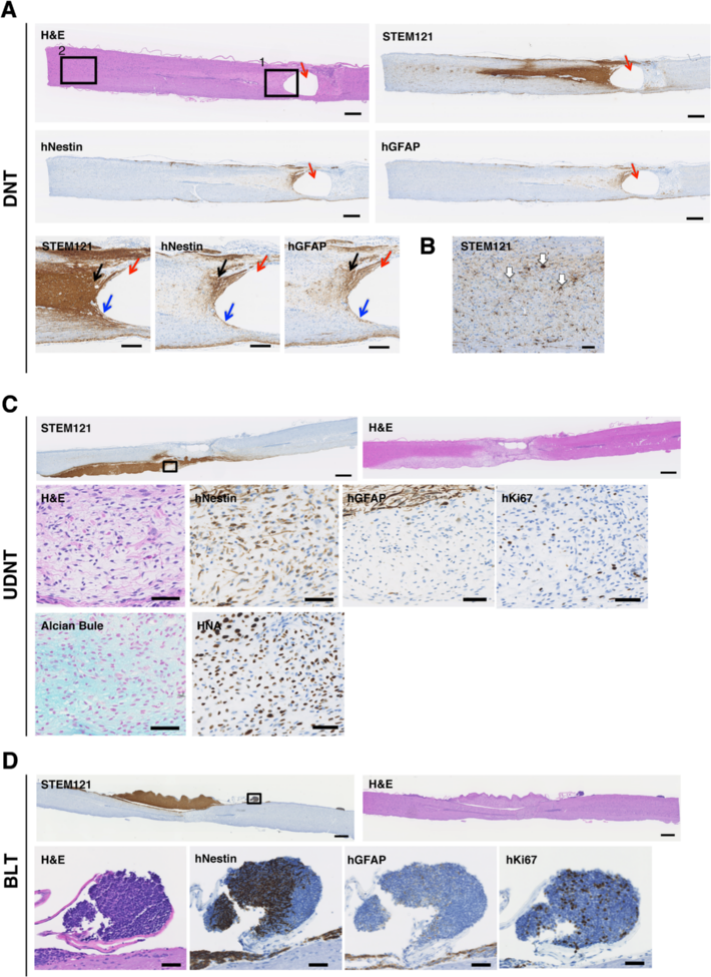
Representative images of DNT, UDNT, and BLT. **a**, **b** Representative images of the DNT with zone formation around the central canal resembling cysts, which were observed in the 1231A3 NR-NSPC transplanted spinal cords 6 months after transplantation. **a** The red arrows indicate the cyst, blue arrows indicate the VZ, and black arrows indicate the IZ. Upper panel: H&E, STEM121, hNestin, and hGFAP. (Scale = 500 μm.) Lower panel: STEM121, hNestin, and hGFAP. (Captured in boxed area 1 in the first panel. Scale = 100 μm.) (**b**) STEM121+ scattered neural cells (SNCs) observed in the MZ that formed in the rostral end of boxed area 2 in Fig. 5A are indicated by white arrows. There were many more migrating neural cells than is indicated. (Scale = 50 μm.) (**c**) Representative histologic features of the UDNT, as observed in a 1201C1 EB-NSPCs transplanted spinal cord at the 12th week after transplantation. Upper panel: STEM121 and H&E. (Scale = 500 μm.) Lower panel: H&E, hNestin, hGFAP, hKi67, Alcian Blue, and HNA. (Captured in the boxed area in the upper panel. Scale = 50 μm.) (**d**) Representative histologic features of the BLT as observed in a 1210B2 NR-NSPC transplanted spinal cord at the 12th week after transplantation. The low power field views of the STEM121 staining is the same image in Fig. 3b. Upper panel: STEM121 and H&E. (Scale = 500 μm.) Lower panel: H&E, hNestin, hGFAP, and hKi67. (Captured in the boxed area in the upper panel. Scale = 50 μm.)

Following the characterization of NPSC proliferative capacities in vitro and in vivo, it remained unclear how iPSC-derived NSPCs differentiate, distribute, and contribute to host tissue. Assessment of the tumorigenicity of iPSC-NSPCs-derivatives is also an important goal. To clarify and classify the histology of the transplants in developmental terms, we also transplanted other NSPC lines [20, 23, 24] and performed histochemical analysis (Table 1 and Figs. 4 and 5). For a comparator, we used long-term self-renewing neuroepithelial-like stem cells (lt-NESCs) [20, 24, 25] to determine whether cells cultivated in an adhesive culture in vitro behave differently in vivo. Additionally, we also used fetal-derived NS/PCs [23] as a control that does not malignantly transform, based on previous observations. As a result, although the transplanted iPSC-derived NSPCs and fetal-derived NSPCs mainly differentiated into neural and rarely mesenchymal tissues, tissue maturity was closely associated with the sites to which transplanted cells terminally migrated (Table 1A, B).

**Table 1:** Classification of the tissue histology formed by iPSC-NSPCs in mouse CNS

(A) Histology classification of tissues with maturational patterns.			
Differentiation	Neural differentiation		Mesenchymal differentiation
Maturation	Maturing/matured	Under-maturation	Maturing/matured
Histology (Identification on H&E staining)	DNT (either identifiable or non-identifiable)	BLT (identifiable)	Benign mesenchymal tissue (identifiable)
		UDNT (identifiable)	
Location	DNT: (1) Parencymal DNT; around central canal or cyst (2)Subpial DNT; beneath the pia matter	BLT:Usually extraparenchymal, but rarely found intraparenchymal	Benign mesenchymal tissue: Extraparenchymal
		UDNT: Extraparenchymal and intraparenchymal	
(B) Scattered neural cell classification.			
	Migrating neural cells	Subpial neural cells	
Maturation	Immature/maturing (NESTIN+/-)	Immature/maturing (NESTIN+/-)	
	Mature (NESTIN-)		
Fate	(1) Individual cell maturation	Contribute to subpial DNT formation	
	(2) Contribute to DNT formation		
	(3) Possibly involved in UDNT formation

When graft-derived cells were found in the vicinity of the central canal or the pia mater, they often formed neural differentiated tissues, which were further classifiable into two groups according to their maturation level (Table 1A and Figs. 4A, B and 5a, c, d): (1) a well-maturing/matured group, termed differentiating neural tissue (DNT), and (2) an under-matured group, which we termed undifferentiated neural tissue (UDNT), and blastemal tissue (BLT). Scattered transplanted cells that reached neither the central canal nor the pia mater were defined as migrating neural cells, which were also classifiable into immature/maturing and mature subtypes (Table 1B and Fig. 5b).

The tissue pattern, which we named DNT, typically consisted of three different maturational stages of neural tissue that showed sequential differentiation that closely resembled zone formation in fetal spinal cord or brain development (Fig. 5a). More specifically, cells that were only Nestin-positive appeared to migrate into the ependymal cell layer beneath the central canal or into the pia mater where it was considered equivalent to the ventricular zone (VZ). Of these cells, neuroepithelium-like or subependymal cell-like neural stem cells differentiated into glial and neuronal lineages and formed an area histologically similar to the intermediate zone (IZ) (Figs. 4A, B and 5a). The IZ-equivalent area subsequently transited to a cell-poor area that consisted mostly of fibers distal from the VZ zone, which we considered equivalent to the marginal zone (MZ) (Figs. 4A, B and 5a). In some cases, the DNT was not identifiable by H&E staining, as it intermingled well with the pre-existing host nerve tissue, which supported its successful maturation. Additionally, the DNT could be further classed into two subtypes based on the terminal destinations of the transplanted stem cells: a parenchymal (conventional) subtype, which arose from subependymal areas of the central canal, and a subpial subtype, derived from cells that attached to the pia mater.

The tissue patterns, which were referred to as undifferentiated neural tissue (UDNT) and blastemal tissue (BLT), were under-matured and exhibited incomplete and arrested differentiation of nervous tissues, respectively, which were frequently found outside the spinal cord or brain parenchyma (Fig. 4A, B). The UDNT was typically composed of incompletely differentiated neural cells within a myxomatous background and were characterized by Nestin expression and the absence or faint expression of GFAP (Fig. 5c). The UDNT was thought to originate from blastemal cells and to be able to partially differentiate into mature neural cells. BLTs were observed as an aggregation of cells with an embryonic appearance with or without neural processes; these cells positively or negatively expressed Nestin, indicating remnants of the primitive neural cells (Fig. 5d). BLT in the meninges tended to extend in a rostro-caudal direction. We observed that BLT is able to differentiate into UDNT, as transition foci from blastemal cells to UDNT were occasionally found. We also confirmed BLT maturational capacity by comparing the spinal cord histology at weeks 12 and 26 post-human-fetal NSPC transplant (Additional file 10: Figure S4A, S4B). Interestingly, the BLT in the meninges rarely differentiated into benign mesenchymal tissues (Additional file 10: Figure S4C). These mesenchymal tissues may be the result of contamination by cells from other lineages, such as neural crest stem cells (NCSCs), indicating that BLT histology possibly includes cells of both neural and mesenchymal lineages.

Among the animals analyzed, no mice developed malignant tumors or teratomas derived from the transplanted cells. Furthermore, no mice that received transplants to injured spinal cord showed evident paralysis from the proliferation of the transplants, as observed in a previous report [14]. There was a tendency for spinal cord-injured mice to show weakness in the lower limbs after 5 months; however, this may reflect a more general tendency of the mice used, as not all of them with weakness had large tissue formations from the transplants, and the life expectancy of NOD/*scid* mice is estimated at ~8 months (Additional file 11: Figure S5A, S5B).

After comparing the in vitro data with the in vivo histological classifications of the six NSPC lines produced for this study (Table 2), the 1231A3 EB-NSPCs with the CNV abnormalities tended to remain in an immature state. However, the 1231A3 NR-NSPCs, which had larger CNV abnormalities and also karyotype abnormalities, or the 1201C1 NSPCs, which also had some CNV abnormalities, did not show apparent immature phenotypes. These results indicate that the cellular CNV or karyotype-only abnormalities do not explain the maturation arrest of the transplants in vivo. Rather, transplant maturation was affected by the terminal destination tissue to which transplanted cells migrated.

**Table 2:** Comparison of histological classifications and the abnormalities observed in the in vitro analysis of each iPSC-NSPC line

NSPCs	Abnormality in in vitro analysis	Number of animals for each experiment (histology taken at 3 months/ 6 months)	Percentage of neural differentiation seen in each experiment			
			(histology taken at 3 months/6 months)			
		Spinal cord	Graft failure	DNT	UDNT	BLT
		Brain				
1210B2 EB-NSPCs	CNV (no* de novo*, very few additional)	3/2	None	100 %/100 %	33 %/0 %	33 %/0 %
				(*n* = 3/2)	(*n* = 1/0)	(*n* = 1/0)
		3/2	None	100 %/100 %	None	None
				(*n* = 3/2)		
1210B2 NR-NSPCs	CNV (one *de novo*, very few additional)	3/2	33 %/0 %	0 %/ 100 %	33 %/0 %	67 %/0 %
			(*n* = 1/0)	(*n* = 0/2)	(*n* = 1/0)	(*n* = 2/0)
		3/2	None	100 %/100 %	None	None
				(*n* = 3/2)		
1231A3 EB-NSPCs	CNV (many *de novo*, some additional)	2/3	0 %/33 %	100 %/67 %	100 %/67 %	0 %/33 %
			(*n* = 0/1)	(*n* = 2/2)	(*n* = 2/2)	(*n* = 0/1)
		3/3	None	100 %/100 %	67 %/100 %	None
				(*n* = 3/3)	(*n* = 2/3)	
1231A3 NR-NSPCs	Karyotype CNV (many* de novo*, many additional)	3/3	None	67 %/100 %	33 %/0 %	33 %/0 %
				(*n* = 2/3)	(*n* = 1/0)	(*n* = 1/0)
		3/3	None	100 %/100 %	None	None
				(*n* = 3/3)		
1201C1 EB-NSPCs	CNV (some *de novo*, no additional)	3/0	None	100 % /-	33.3 %/-	None
				(*n* = 3/-)	(*n* = 1/-)	
		3/3	None	100 %/100 %	0 %/67 %	None
				(*n* = 3/3)	(*n* = 0/2)	
1201C1 NR-NSPCs	CNV (some *de novo*, no additional)	3/3	33 %/67 %	67 %/33 %	33 %/0 %	None
			(*n* = 1/2)	(*n* = 2/1)	(*n* = 1/0)	
		5/1	None	100 % /100 %	None	None
				(*n* = 5/1)		

### Public registry of histology data and histological diagnoses from this study

To share the large amount of information from our histological sections and the histological diagnoses performed in this study, we registered a set of our organized data to an open-access website (https://www.skip.med.keio.ac.jp/iPSC-NSPC/). High-resolution images of every histological section of the 137 mice used in this study that are not shown in this paper are accessible on this website, which makes it possible to scan across and magnify individual sections of large images. This online archive also contains the precise histological definitions and diagnoses for each section, which we hope will contribute to further discussion of histological derivatives following NSPC transplantation, as well as safety issues and possibilities for clinical applications.

## Discussion

The risk of teratoma formation by transplanted iPSC-derivatives is widely recognized, and many attempts have been made to minimize such risks [26, 27]. Although the transformation of iPSC-derived products has not been studied as extensively, a number of reports have showed the transformation of iPSC-derived intermediate progenitor cells as the result of genetic modification [14, 28, 29], transgene activation [30], or epigenetic events [31]. We used integration-free iPSCs to minimize the risk of genetic modification and/or transgene re-activation, which were observed in a previous report by our group [14]. However, some of the NSPCs from the three integration-free iPSCs used in this study retained proliferative characteristics in vitro and in vivo, which appeared to be attributable to karyotype abnormalities or *de novo* CNVs that occurred during differentiation and culture processes. As the three iPSC-lines utilized in the present study were induced from blood samples from a single donor, the different genomic abnormalities observed in our iPSC lines may have occurred either during the reprogramming process or in cell culture. This suggests that even when integration-free iPSCs are used, it is not possible to completely eliminate the risk of genetic instability during NSPC production. This also indicates that the neural induction protocols that we used cannot eliminate all cells with genetic instability, and that genetic differences that emerged during the reprogramming process from the original iPSCs cannot be fully standardized. To control the proliferation of the derivatives in vivo, we thus should use iPSC lines without karyotype abnormalities or genomic instabilities.

Culture duration is also important in controlling the genomic and epigenomic character of cultured cells. Young iPSCs may be immature and unstable, but it has also been reported that culture-induced genomic and epigenomic aberrations can occur at any stage, as the undifferentiated state of iPSCs is inherently unstable and sensitive [32]. We decided to use iPSCs as early as 11 passages for neural induction from feeder-free cultured iPSCs (1210B2 and 1231A3), in order to lower the risk of genetic abnormality associated with long-term culture process. Induced pluripotent stem cells established using the same method have been reported to express pluripotency-markers by passage 5 [33]. We also confirmed expression of pluripotent markers in our cells by passage 11. Additionally, we needed to cultivate the cells for this length to obtain a sufficient number of cells for further use. With respect to on-feeder iPSCs (1201C1), we needed to culture these until passage 19 in order to obtain sufficient numbers of cells. The adequate passage numbers of iPSCs or NSPCs may thus differ depending on the culture method and the purpose of usage of cells, a condition that should be evaluated for each case.

It has been reported that iPSCs tend to acquire epigenetic and genetic modifications, including CNVs, during the reprogramming process and subsequent cell culture [34–36], and that these genetic alterations may occur in genomic sites related to cancer development. It is impossible to eliminate the risk of genetic abnormality; however, our results with the 1210B2 EB- and NR- NSPCs indicate that the proliferative capacity of the NSPCs can be lowered by selecting cells with few genetic abnormalities. Careful assessment of cells, including their genomic stability, may thus be useful for reducing risks in transplantation.

Considering the differentiation of the induced NSPCs in vivo, the differentiation tendency was affected by the induction protocols used. It has also been reported that cellular migration or survival is affected by the injection site, timing of the transplant after injury, as well as by transplant dose [37–39]. These properties are relevant to the normal mammalian CNS developmental process. During mammalian CNS development, NSPCs gain temporal and positional identities, and thus acquire defined and limited plasticity, such as neurons or glial cells [40, 41]. We categorized the maturation of transplanted NSPCs by their formation of different developmental tissues, and found that the environment of the cells’ terminal destination site affected their developmental fate. When transplanted cells engrafted outside the CNS parenchyma, they nearly always underwent maturational arrest or delay of neural tissues, which were represented by BLT and UDNT. BLTs are the most primitive tissues, and when they were rarely found within the parenchyma, they exhibited extensive neural differentiation and maturation characteristics (Additional file 9: Figure S3A, S3B). In support of these developmental events, transplanted cells within the parenchyma of the CNS often formed neural tissues with adequate maturation (DNT).

These results indicate that the control of cellular migration is useful in controlling the fate of transplants. Considering the potential for clinical application of NSPC transplantation, the preferable histology would be different according to each purpose. Animal models with environments similar to those of recipient patients will be most informative with respect to the engraftment style and the migration potential of the NSPCs. However, in addition to NSPC transplantation using animal models that precisely reflect the disease environment in clinical settings, other conventional animal models may be used for specific purposes. For example, when assessing NSPCs to be used in the development of SCI treatments, we should select animal models of spinal cord injury to evaluate cellular maturation stages. Transplantation into intact animal brain, which is much easier and enables the assessment of larger numbers of cells at a given time, would also be informative in the assessment of the proliferative capacity of transplanted cells, as the proliferative tendencies of each cell line were similar in the intact brains and injured spinal cords evaluated in our study.

Interestingly, it appeared that neural tissues were regularly generated in developmental stages from particular regions (e.g., central canal, central canal-like cyst, and pia mater), particularly in injured spinal cords, whereas they frequently formed and grew in a round shape without associations with ventricles in non-pathologic brains. Other studies also indicate that cells proliferate better under pathologic conditions [38, 42]. We speculate that under pathologic conditions, transplanted cells may favor migration into appropriate areas from which tissue or organ regeneration can be initiated, as is observed in fetal development, thus resulting in better engraftment.

The aim of the present study was to classify the histology of transplanted human iPSC-NSPCs in the mouse CNS by developmental potential to aid in the identification of malignant transformations of transplants during safety assessment of cells intended for use in transplantation therapy. Distinguishing between embryonic tissues and tumors by microscopy is difficult, as normal developing tissues in which stem cell multiplication occurs have significant mitotic activity similar to that of malignant tumors. Furthermore, in humans, especially in children, it is known that tumors often arise from remnants of embryonal tissues; however, the majority of such tissues involute or differentiate into mature tissues [43].

The definition of a tumor is a mass of cells that proliferate without relation to pattern or rate of the growth of the part in which it is located. This unlimited cellular growth may lead to the development of malignant properties, including invasion of surrounding tissues and metastasis to other organs [44]. In this study, we concluded from the following observations that none of the transplanted cells developed into malignant tumors. 1) We observed that the transplanted cells generated nervous tissues with differentiation regularity and limited growth, which we interpreted as a recapitulation of zonal formation in CNS development, as represented by characteristic DNT features. 2) Some UDNTs showed extensive growth along the meninges. This may suggest that these cells have unlimited proliferation potential and immature properties. However, the margin of this tissue was smooth and tended toward neural maturation, and no invasion of adjacent tissues has been observed so far. We thus classed this histologic subtype as tissue overgrowth accompanying cellular immaturity. 3) BLT induces the emergence of embryonal tumors, such as neuroblastomas, medulloblastomas, primitive neuroectodermal tumors, and Wilms tumors. The BLTs observed in the current study were a confined tissue that was occasionally found in the meninges. Additionally, BLT lacks mitotic figures and can differentiate into neural and mesenchymal lineages. These features may more closely resemble perilobar nephrogenic rests (PLNR), which are embryonal tissue remnants observed in kidneys of patients with overgrowth syndrome [45]. BLT may differentiate into mature tissue or regress, as is observed in PLNRs. Only a few PLNR types develop into Wilms tumors [46].

Considering the implications for these observations in human, we predict that if transplanted iPSC-derived cells can become cancer cells, they would be BLT-derived embryonal tumor cells that tend to localize in the meninges rather than an adult cancer type. We did not observe any cancerous lesions in the present study, but recognize that any cell has the potential to develop into cancer. Longer-term observation is thus required to determine ways in which malignant tumorigenesis can be inhibited.

Several approaches to the minimization of transplantation risk have been reported. In addition to eliminating pluripotent cell contamination, controlling the proliferative characteristics of cells by pre-treating them with MitomycinC [47] or γ-secretase [48] may be effective. Transplant ablation by transgenic *HSV-tk* and gancyclovir administration [49], or a caspase-based artificial cell death switch (iCaspase-9) [50] may also be effective in cases of malignancy. In all cases, the correct diagnosis of acceptable or unacceptable histology must be made. From this perspective, our histological classification is also an effective approach for optimizing the safety of NSPC transplants.

These results provide important histological insights into the transplantation of human NSPCs into the CNS of animal models, with a focus on safety issues confronting future cell transplant therapeutics.

## Methods

Additional details regarding several of the protocols used in this work are provided in the Additional files 12, 13, 14 and 15: Supplemental Experimental Procedure and Tables S7-S9.

### Cell culture

Three lines of integration free human PBMC-derived iPSCs (1210B2, 1231A3, and 1201C1), which were established from ePBMCs® from the Cellular Technology Limited (OH, USA) at Center for iPS Cell Research and Application (CiRA: Kyoto, Japan) by an integration-free method [51], were used. The 1210B2 and 1231A3 iPSCs were cultured with a feeder-free protocol [33], and the 1201C1 iPSCs were cultured with an on-feeder protocol that uses SNL feeder cells. They were induced into NSPCs as previously described [52, 53] with two slight modifications. Briefly, in the first protocol, the NSPCs were induced directly from embryoid bodies (EBs) by a protocol that consists only of a floating culture. In the second protocol, the EBs were adhered to laminin-coated culture dishes on day 7, and they subsequently formed neural rosettes (NRs), which were picked on day 14. We refer to NSPCs induced directly from EBs as EB-NSPCs, and those induced from the NR phase as NR-NSPCs. The NSPCs were expanded using the neurosphere culture technique [23, 54].

### Cellular analysis

Detailed experimental procedures were described in the supplemental materials. For the microarray analysis, total RNA was analyzed using Human Genome U133 Plus 2.0 Arrays (Affymetrix Inc., Santa Clara, CA) according to the manufacturer’s instructions. RT-PCR analysis was performed, as described previously [54]. Cell surface marker expression was analyzed with a BD FACS Verse (BD Biosciences, San Jose, CA). For cell cycle analysis, the DNA contents of the cells were analyzed by propidium iodide staining with an EC800 Analyzer (Sony Biotechnology Inc., Tokyo, Japan). The proliferation assay was performed by measuring ATP with a CellTiter-Glo Luminescent Cell Viability Assay (Promega, Madison, WI) on an ARVO X5 Multilabel Plate Reader (PerkinElmer, Waltham, MA). The cellular doubling time was calculated from the intensities of two sampling points in the logarithmic growth phase, as previously shown [23]. The karyotype analysis was performed by conventional Giemsa staining and G-band analysis, and diagnosed as issued in the 2013 international system for human cytogenetic nomenclature (ISCN 2013) [55]. CNV was analyzed using a CytoScan HD Array (Affymetrix Inc., Santa Clara, CA) according to the manufacturer’s instructions. For NSPC differentiation, cells were plated on Matrigel (Corning) and cultured in the same medium, supplemented with 1 % fetal bovine serum instead of growth factors. Phenotype analysis of differentiated cells was performed using immunocytochemistry on an IX81 microscopy with a fluorescence module (Olympus Corp., Tokyo Japan).

### Animal model and cellular transplantation

All mouse studies were conducted in strict accordance with the Guide for Care and Use of Laboratory Animals of the Central Institute for Experimental Animals (CIEA, Kanagawa, Japan), and the experimental protocols were approved by the CIEA Animal Care Committee (Permit Number: 11029A) in accordance with Keio University School of Medicine (Tokyo, Japan) (Permit Number: 16-096-25).

For the brain transplant model, NSPCs were injected bilaterally into the striata of 9 week-old female NOG mice (2.0×10^6^ cells per mouse) (Clea Japan, Tokyo, Japan). For the spinal cord injury models, the cell transplantations were performed on 9 week-old female NOD/*scid* mice (Charles River Laboratories Japan, Inc., Tokyo, Japan), as previously described [14]. Briefly, 5×10^5^ NSPCs were transplanted to the epicenter of the injury 9 days after the moderate contusion injury (IH impactor, 60-70kdyn). After the transplantation, the brain transplant models were monitored for abnormal behavior, and the spinal cord injury models were monitored for lower limb motor function using the Basso Mouse Scale (BMS) [56].

The mouse strain type differed between the transplantation models because of the capacity lamination of our facility to harvest immunodeficient animals.

### Histological Analysis

Twelve to 26 weeks after transplantation, mice were sacrificed, and their brains or spinal cords were taken to make paraffin sections, which were evaluated via H&E staining or immunohistochemistry. In all the sagittal spinal cord sections, the left side indicates the rostral and the upper side indicates the dorsal side of the section.

The extent of the transplants in the CNS of the transplanted animals was analyzed by measuring the STEM121-positive and -negative areas using Adobe Photoshop (version 13.0; San Jose, CA, USA).

To produce pathological classifications, additional histology data from transplantation of human fetal NSPCs and other iPSC-derived NSPCs were evaluated.

### Statistics

A significance criterion of *p* < 0.05 was used. A nonparametric Kruskal-Wallis test followed by the Mann-Whitney *U* test were used to analyze RT-PCR and graft volumes.

## Additional files

Additional file 1: Figure S1. Supplemental 1231A2-NSPC and 1201C1-NSPC data for Fig. 1b, c, and d. (A) FACS analysis results. (B) RT-PCR analysis results. (C) In vitro differentiation assay immunocytochemical data results. (Scale = 100 μm.). (TIF 3241 kb) Additional file 2: Table S1. Gene expression profile correlation coefficients. (XLSX 46 kb) Additional file 3: Table S2. Karyotype analysis results of NSPCs using conventional giemsa staining and G-banding. (XLSX 43 kb) Additional file 4: Figure S2. Supplemental CNV analysis data. Complete CNV data for the performed whole genome view. (TIF 2632 kb) Additional file 5: Table S3. Summary of the in vitro *de novo* copy number variations (CNVs) in the NSPCs. (A) Total numbers of the *de novo* CNVs in NSPCs during neural differentiation and neurosphere culture by passage 7. (B) Numbers of the CNVs in NSPCs additionally identified after additional 5 passages of neurosphere culture. (XLSX 36 kb) Additional file 6: Table S4.
*De novo* copy number variations (CNVs) identified in the 1210B2 iPSC-derived NSPCs. (XLSX 39 kb) Additional file 7: Table S5.
*De novo* copy number variations (CNVs) identified in the 1231A3 iPSC-derived NSPCs. (XLSX 79 kb) Additional file 8: Table S6.
*De novo* copy number variations (CNVs) identified in the 1201C1 iPSC-derived NSPCs. (XLSX 35 kb) Additional file 9: Figure S3. Representative tissue sections of the spinal cord (upper row, 12 weeks after transplant) and brain (lower row, 12 weeks after transplant) after the transplantation of AF22. Immunohistochemistry results for STEM121 and DAB, which were positive in the cytoplasm of the transplanted human cells. (Scale = 500 μm.). (TIF 2126 kb) Additional file 10: Figure S4. Representative images of transplant-derived tissue from human embryo-derived NSPCs and NSPCs with portions of NCSCs. (A) An injured spinal cord 12 weeks after transplantation with human embryo-derived NSPCs. The red arrow indicates the hGFAP negative BLT. Upper panel: H&E and STEM121. (Scale = 500 μm.) Lower panel: H&E, hNestin, hGFAP, and Ki67. (Captured in the boxed area in the upper panel. Scale = 100 μm.) (B) An injured spinal cord 26 weeks after transplantation with the same NSPCs used in Additional file 9: Figure S3A. Most of the blastemal features of the transplants observed at week 12 disappeared by week 26, which shows the maturational capacity of the BLT. Upper panel: H&E and STEM121. (Scale = 500 μm.) Lower panel: H&E, STEM121, and hGFAP. (Captured in the boxed area in the first panel. Scale = 100 μm.) (C) Representative histology of mesenchymal tumors derived from 1210B2-ltNESC. Here, the transplanted NSPCs had some NCSC contamination. The section evaluated 26 weeks after transplantation revealed transplant-derived bone formation in the meningeal space. Upper panel: H&E. (Scale = 500 μm.) Lower panel: H&E and STEM121 (Captured in the boxed area in the first panel. Scale = 100 μm.). (TIF 7362 kb) Additional file 11: Figure S5. Open field scores for mice with transplantations in their injured spinal cords. (A) Every mouse with a spinal cord injury was evaluated for lower limb motor function. The red lines indicate mice with histology shown in Fig. 3b. *A mouse that had its histology used in Fig. 5a. **Mice with weakness at the end of the observation period. (B) STEM121 immunostaining of their spinal cords at week 26 shows that the weakness did not always accompany a large lesion that was occupied by the transplants. (TIF 4013 kb) Additional file 12: Supplemental experimental procedures. (DOCX 25 kb) Additional file 13: Table S7. Primer information (XLSX 35 kb) Additional file 14: Table S8. Antibodies utilized for flow cytometry (XLSX 37 kb) Additional file 15: Table S9. Antibodies utilized for immunostaining (XLSX 43 kb)

## Abbreviations

BLT: Blastemal tissue
CNS: Central nervous system
CNVs: Copy number variations
DNT: Differentiating neural tissue
EB: Embryoid body
iPSC: induced pluripotent stem cell
IZ: Intermediate zone
lt-NESCs: Long-term self-renewing neuroepithelial-like stem cells
MZ: Marginal zone
NCSCs: Neural crest stem cells
NR: Neural rosette
NSPC: Neural stem progenitor cell
PBMC: Peripheral blood mononuclear cell
UDNT: Undifferentiated neural tissue
VZ: Ventricular zone
