# Supplementary figures and images for: Pathological classification of human iPSC-derived neural stem/progenitor cells towards safety assessment of transplantation therapy for CNS diseases
Source: Mol Brain. 2016 Sep 19;9:85. doi: 10.1186/s13041-016-0265-8 (PMC5027634; doi:10.1186/s13041-016-0265-8)

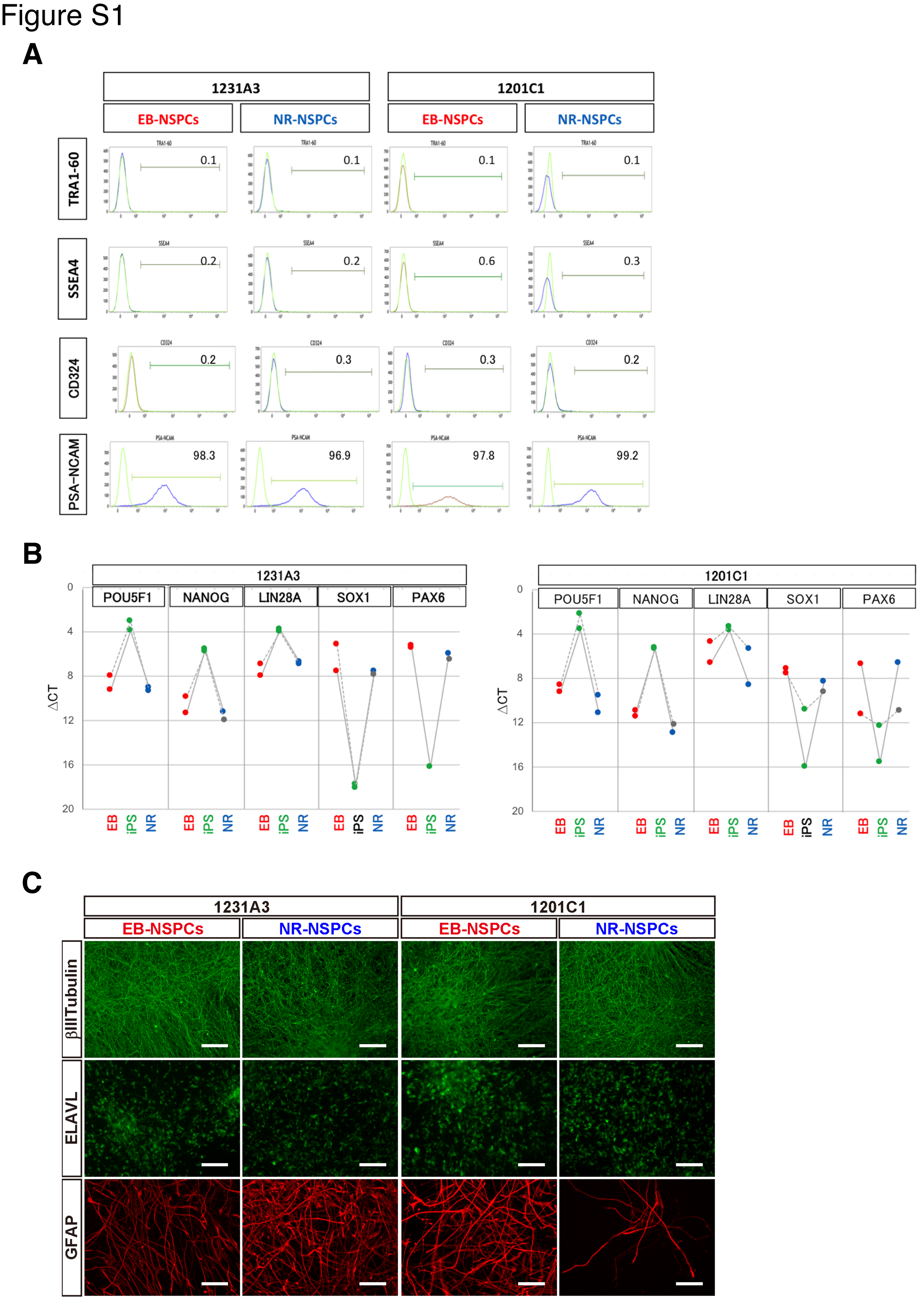

Supplement: Additional file 1: Figure S1. — Supplemental 1231A2-NSPC and 1201C1-NSPC data for Fig. 1b, c, and d. (A) FACS analysis results. (B) RT-PCR analysis results. (C) In vitro differentiation assay immunocytochemical data results. (Scale = 100 μm.). (TIF 3241 kb) [file 13041_2016_265_MOESM1_ESM.tif]

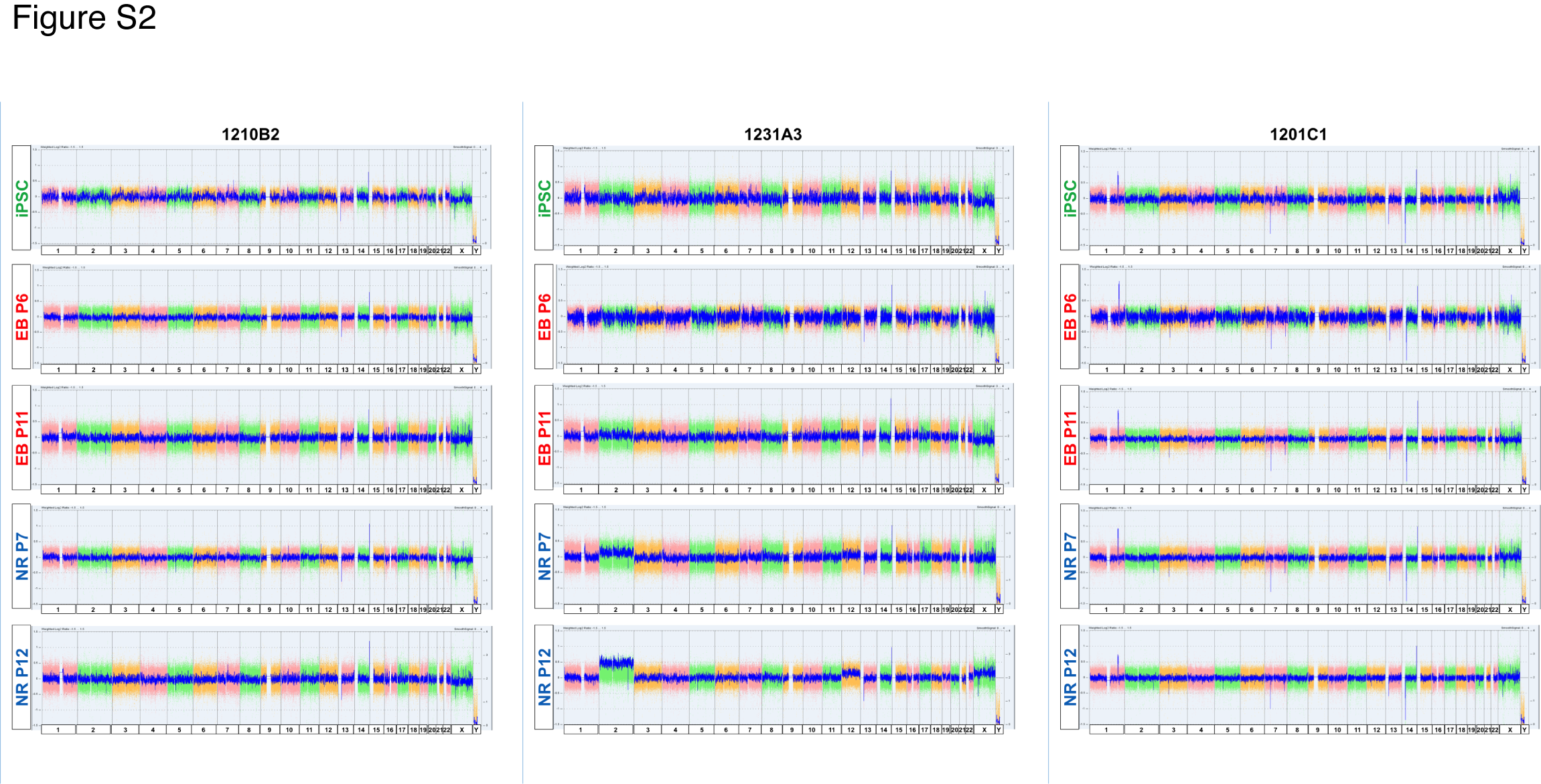

Supplement: Additional file 4: Figure S2. — Supplemental CNV analysis data. Complete CNV data for the performed whole genome view. (TIF 2632 kb) [file 13041_2016_265_MOESM4_ESM.tif]

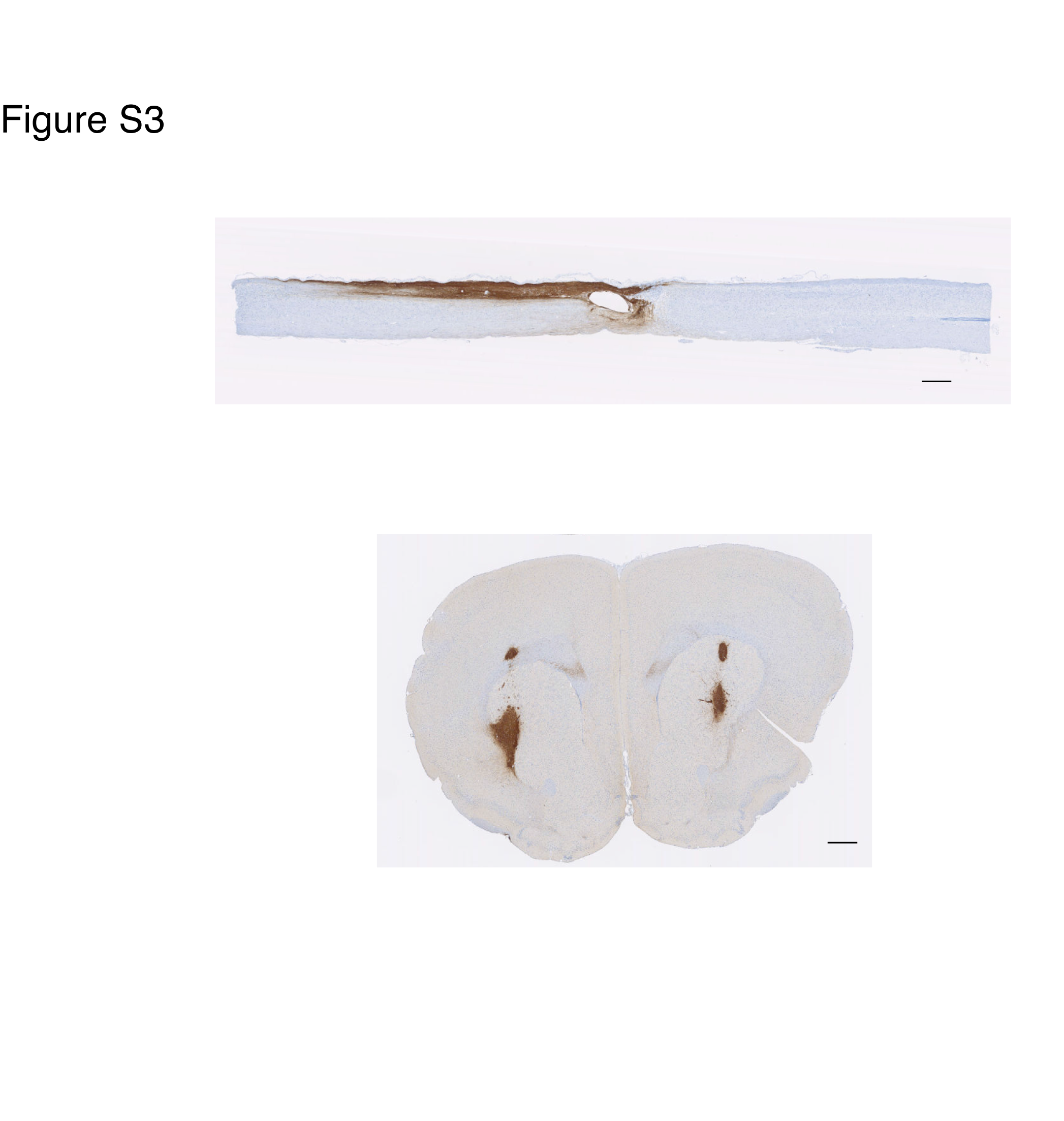

Supplement: Additional file 9: Figure S3. — Representative tissue sections of the spinal cord (upper row, 12 weeks after transplant) and brain (lower row, 12 weeks after transplant) after the transplantation of AF22. Immunohistochemistry results for STEM121 and DAB, which were positive in the cytoplasm of the transplanted human cells. (Scale = 500 μm.). (TIF 2126 kb) [file 13041_2016_265_MOESM9_ESM.tif]

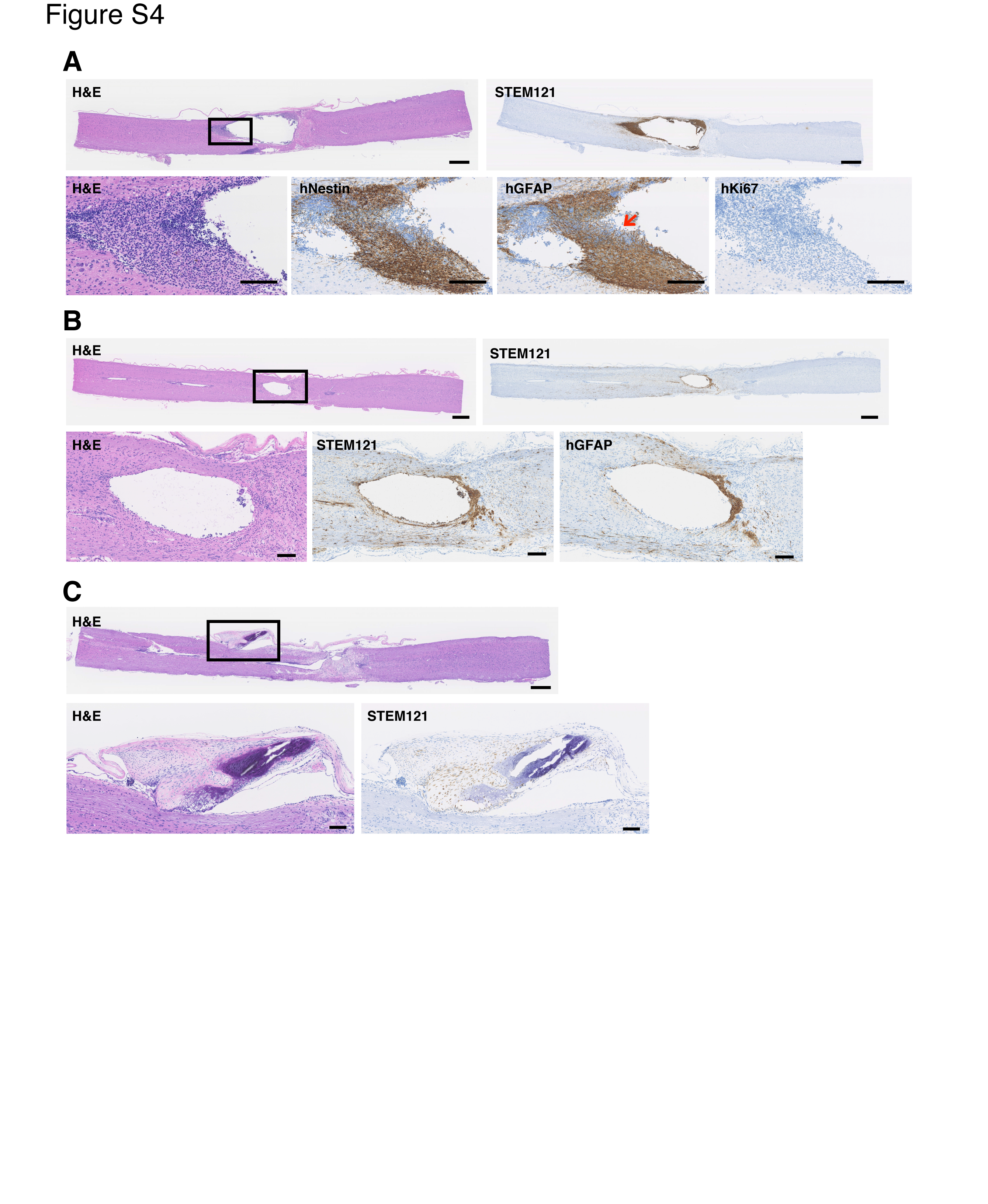

Supplement: Additional file 10: Figure S4. — Representative images of transplant-derived tissue from human embryo-derived NSPCs and NSPCs with portions of NCSCs. (A) An injured spinal cord 12 weeks after transplantation with human embryo-derived NSPCs. The red arrow indicates the hGFAP negative BLT. Upper panel: H&E and STEM121. (Scale = 500 μm.) Lower panel: H&E, hNestin, hGFAP, and Ki67. (Captured in the boxed area in the upper panel. Scale = 100 μm.) (B) An injured spinal cord 26 weeks after transplantation with the same NSPCs used in Additional file 9: Figure S3A. Most of the blastemal features of the transplants observed at week 12 disappeared by week 26, which shows the maturational capacity of the BLT. Upper panel: H&E and STEM121. (Scale = 500 μm.) Lower panel: H&E, STEM121, and hGFAP. (Captured in the boxed area in the first panel. Scale = 100 μm.) (C) Representative histology of mesenchymal tumors derived from 1210B2-ltNESC. Here, the transplanted NSPCs had some NCSC contamination. The section evaluated 26 weeks after transplantation revealed transplant-derived bone formation in the meningeal space. Upper panel: H&E. (Scale = 500 μm.) Lower panel: H&E and STEM121 (Captured in the boxed area in the first panel. Scale = 100 μm.). (TIF 7362 kb) [file 13041_2016_265_MOESM10_ESM.tif]

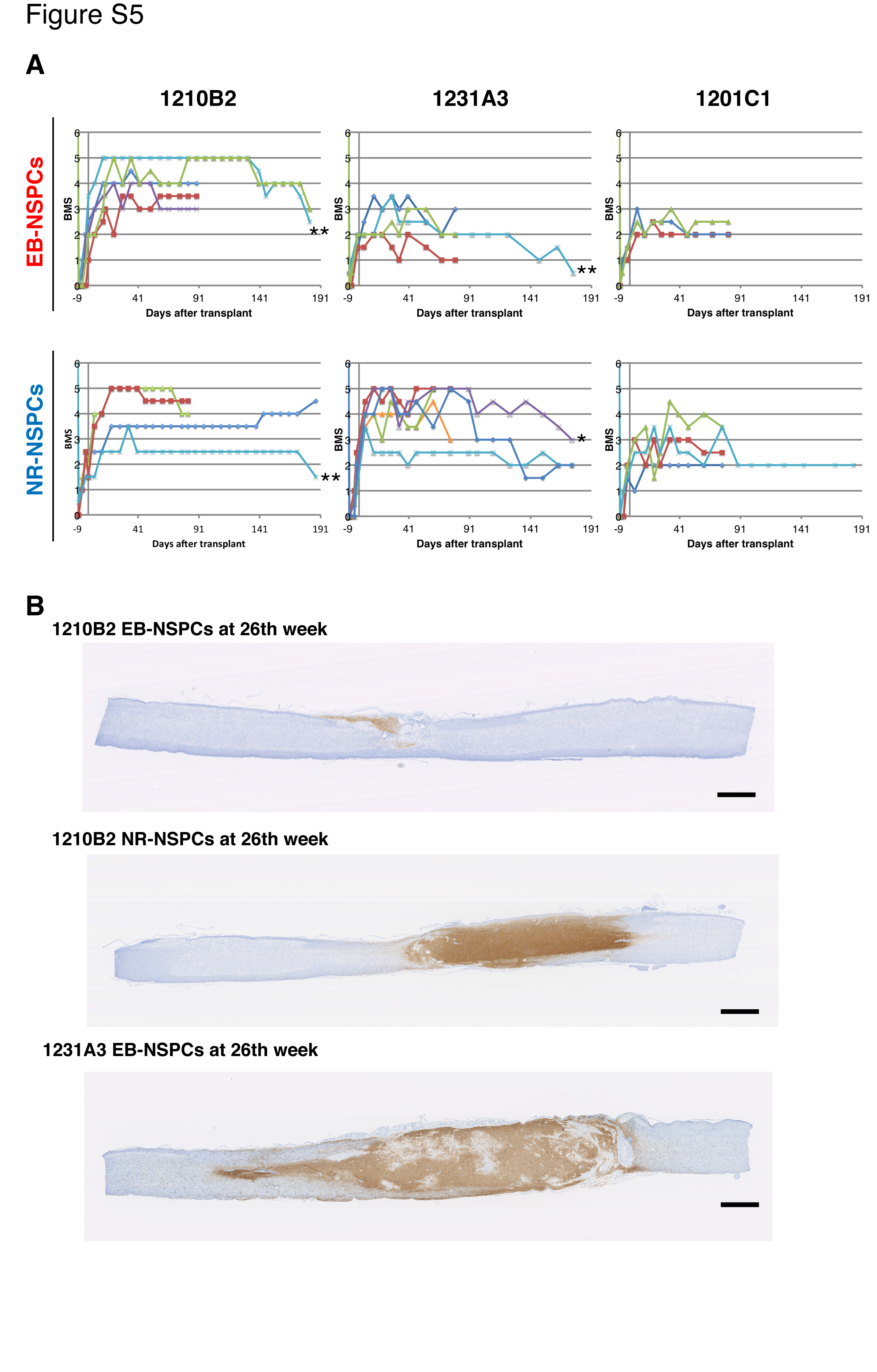

Supplement: Additional file 11: Figure S5. — Open field scores for mice with transplantations in their injured spinal cords. (A) Every mouse with a spinal cord injury was evaluated for lower limb motor function. The red lines indicate mice with histology shown in Fig. 3b. *A mouse that had its histology used in Fig. 5a. **Mice with weakness at the end of the observation period. (B) STEM121 immunostaining of their spinal cords at week 26 shows that the weakness did not always accompany a large lesion that was occupied by the transplants. (TIF 4013 kb) [file 13041_2016_265_MOESM11_ESM.tif]
