# Supplementary material for: Pathological classification of human iPSC-derived neural stem/progenitor cells towards safety assessment of transplantation therapy for CNS diseases
Source: Mol Brain. 2016 Sep 19;9:85. doi: 10.1186/s13041-016-0265-8 (PMC5027634; doi:10.1186/s13041-016-0265-8)
Supplement: Additional file 12: — Supplemental experimental procedures. (DOCX 25 kb) [file 13041_2016_265_MOESM12_ESM.docx]

**Supplemental Experimental Procedures**

**Cell Culture**

1210B2 and 1231A3 iPSCs were maintained on iMatrix-511 (0.5 µg/cm2; Nippi Inc., Tokyo, Japan) -coated cell culture plates with StemFit®AK01 media (Ajinomoto Co. Inc., Tokyo, Japan). 1201C1 iPSC was cultured on mitomycin C-treated SNL feeder cells with Primate ES medium (ReproCELL Inc., Kanagawa, Japan). Neural differentiation was performed using quick-aggregation procedure of serum-free culture of embryoid body-like aggregates [SFEBq] protocol (EB-NSPCs; Kim et al. 2010 and Morizane et al. 2013), and SFEBq followed by neural rosette formation protocol (NR-NSPCs; Zhang et al. 2001). After neural induction of 14 days, both NSPCs were transferred and expanded using the neurosphere culture technique (Kanemura et al. 2002) in DMEM/F-12 (1:1; Sigma-Aldrich, St Louis, MO), with epidermal growth factor (20 ng/ml; PeproTech Inc., Rocky Hill, NJ), fibroblast growth factor 2 (20 ng/ml; PeproTech), leukemia inhibitory factor (10 ng/ml; Millipore Corporation, Billerica, MA), B27 supplement (final 2%; Thermo Fisher Scientific, Inc., Grand Island, NY), and heparin (5 mg/ml; Sigma-Aldrich).

**RNA analysis**

Total RNAs were isolated from iPSCs and NSPCs by QIAzol Lysis Reagent (Qiagen, Valencia, CA, USA). For microarray analysis, 100 ng of total RNA was analyzed by Human Genome U133 Plus 2.0 Array (Affymetrix Inc., Santa Clara, CA) according to the manufacturer’s instructions. Array data were analyzed using Partek Genomic Suite (Partek Inc., St. Louis, MI). For quantitative RT-PCR analysis was performed as described previously (Shofuda et al. 2012). Briefly, cDNA was synthesized by PrimeScript® RT reagent Kit, according to the manufacturer’s specification (Takara Bio, Shiga, Japan). Quantitative PCR analysis was performed using gene-specific primers (Table S7) with Power SYBR® Green PCR Master Mix, and QuantStudio™ 12K Flex Real-time PCR system (Applied Biosystems, Foster, CA). Gene expression was quantitated using comparative Ct method.

**Cytometric analyses**

Cytometric analyses were performed after preparation of single-cell suspensions by TrypLE Select Enzyme (Thermo Fisher Scientific Inc.). Expression of cell surface markers were analyzed by BD FACSVerse (BD Biosciences, San Jose, CA), after reaction with antibodies for 30 min at room temperature (Table S8). For cell cycle analysis, cells were reacted with propidium iodide solution (final concentration: 10 µg/mL) to stain double stranded DNA, and analyzed by EC800 Analyzer (Sony Biotechnology Inc., Tokyo, Japan).

**Cell proliferation analysis**

The proliferation assay was performed by measuring ATP derived from metabolically active cells using CellTiter-Glo Luminescent Cell Viability Assay (Promega, Madison, WI) according to manufacturer’s instruction. Viable cells were measured as luminescence intensity using ARVO X5 Multilabel Plate Reader (PerkinElmer, Waltham, MA) at day 0 and day 3 to 7. Doubling time was calculated from intensities of two sampling point in logarithmic growth phase (Kanemura et al., 2002).

**Karyotype analysis**

Karyotype analysis was performed by conventional Giemsa staining and G-band analysis, and diagnosed as issued in an international system for human cytogenetic nomenclature 2013 (ISCN 2013). Briefly, in conventional Giemsa staining, 50 cells were analyzed and every abnormality was counted. G-band analysis was performed with the specimen assayed by GTG-method and cells were analyzed until the number of cells with normal karyotype reached 20. Abnormal clones were defined only when same abnormality was found both in conventional Giemsa staining and G-band analysis. Abnormality was diagnosed when same chromosomal loss was found in more than three cells, or same conformational abnormality or extra chromosomes were found in more than two cells.

**Copy number analysis**

Copy number variation was precisely analyzed by CytoScan HD Array (Affymetrix). Genomic DNAs were extracted using NucleoSpin® Tissue Kit (Machrey-Nagel, Düren, Germany), and then processed according to manufacturer’s instruction. Scanned data were analyzed by Chromosome Analysis Suite (Affymetrix) with high resolution filter setting, then CNVs in NSPCs were compared to their parental iPSCs.

**In vitro differentiation assay**

For differentiation of NSPCs, cells were plated on Matrigel (Corning, Corning, NY)-coated culture plates, and maintained in the neurosphere medium, supplemented with 1% fetal bovine serum, instead of the three growth factors and heparin for 4 weeks (Kanemura et al., 2002). Phenotype of differentiated cells were analyzed using immunocytochemical analysis. Cells were fixed in 4% paraformaldehyde, blocked with 10% normal goat serum, and then, reacted with anti-βIII-tubulin, human GFAP, and Hu (ELAVL) (Table S9). Phenotypes of differentiated cells were obtained using IX81 microscopy with fluorescence module (Olympus Corp., Tokyo Japan).

.

**Animal Model and Cell Transplantation**

For brain transplant model, adult female NOG mice at 9 weeks old were anesthetized by intraperitoneal injection of somnopentyl (12ml/kg) and inhalation of isoflurane (1-0.5%). After making holes on their skull, NSPCs were injected bilaterally into the striatums of 9 week-old female NOG mice (2.0x10^6^ cells per mouse).

Spinal cord injury models were made as described previously (Nori et al., 2015). Briefly, adult female NOD/*scid* mice at 8 weeks old were anesthetized by intraperitoneal injection of ketamine (100mg/kg) and xylazine (10mg/kg), and contuse injury (IH impactor, 60-70kdyn) was made at 10th thoracic vertebrae level. Nine days later, 5x10^5^ NSPCs were transplanted to the epicenter of the injury.

**Histological analysis**

12 to 26 weeks after the transplant, grafted animals were deeply anesthetized and perfused with PBS followed by 4% PFA in PBS. The dissected brains were dehydrated in 100% ethanols, cleared in xylene, and paraffin-embedded. Brain samples were cut in 5μm-thickness to make coronal sections. Spinal cords were trimmed to 14 mm length (7mm rostral and 7mm caudal) centering the point of transplant, and then sliced to 6μm-thickness to make sagittal sections.

**Measurement of Extent of transplants in the CNS of transplanted animals**

From the sections made, the section with widest area of STEM121 positive area were chosen for each animal. And the percentage of STEM121 positive area occupied in each section was measured using Adobe Photoshop (version 13.0; San Jose, CA, USA).

**Additional histological sections used as reference to make pathological classification**

Histological sections obtained by transplanting following four lines of NSPCs to striatum of NOG mice or injured spinal cord of NOD/*scid* mice, in the same protocol, were used.

1, oh-NSC-3-fb (NSPCs expanded from human embryonic forebrain tissues at 9 gestation weeks) (Kanemura et al., 2002)

2, AF22 (NSPCs induced from iPSCs derived from human adult dermal fibroblast) (A Falk et al., 2012)

3, AF23 (NSPCs expanded from human embryonic hindbrain at 5-7 gestation weeks) (J Tailor et al., 2013)

4, lt-NESCs derived from 1210B2 iPSCs (Isoda et al, in preparation)

5, lt-NESCs derived from 1231A3 iPSCs (Isoda et al, unpublished data)
